# Cognitive Mechanisms Explaining the Relationship Between Post-Traumatic Stress and Post-Traumatic Growth in Survivors of Breast Cancer

**DOI:** 10.3390/curroncol32120666

**Published:** 2025-11-28

**Authors:** Vida Mirabolfathi, Fatemeh Ayoubi, Amirreza Nouri, Alireza Moradi, Laura Jobson, Nazanin Derakshan

**Affiliations:** 1 Department of Cognitive Psychology, Institute of Cognitive Science Studies, Tehran 1658344575, Iran; mirabolfathi_v@icss.ac.ir (V.M.); moradi@icss.ac.ir (A.M.); 2 Turner Institute for Brain and Mental Health and School of Psychological Sciences, Monash University, Melbourne 3800, Australia; laura.jobson@monash.edu; 3 School of Psychology and Clinical Language Sciences, University of Reading, Reading RG6 6UR, UK; 4 The Growth and Resilience in Trauma Centre (GRiT), National Centre for Integrative Oncology (NCIO), Reading RG10 9XQ, UK

**Keywords:** breast cancer, cognitive mechanisms, post-traumatic stress, post-traumatic growth

## Abstract

Background: The ability to derive growth from a traumatic event, such as a cancer diagnosis, can facilitate effective adaptation to the challenges associated with cancer survivorship. Objective: In two studies, we investigated the possible cognitive mechanisms explaining the relationship between post-traumatic stress and post-traumatic growth in female survivors of breast cancer. Specifically, Study 1 examined the role of interpretation bias, and Study 2 examined the role of cognitive restructuring of trauma. Methods: In Study 1, 113 participants completed questionnaires assessing stress- and anxiety-related symptomatology, post-traumatic stress and growth, perceived cognitive functioning, and positive interpretation bias. In Study 2, 117 participants completed questionnaires assessing stress and anxiety-related symptoms, rumination, perceived cognitive functioning, cognitive restructuring of trauma, and post-traumatic stress and growth. Results: In both studies, post-traumatic stress was negatively related to post-traumatic growth. In Study 1, positive interpretation bias explained a significant amount of variance in the relationship between post-traumatic stress and post-traumatic growth, with perceived cognitive functioning moderating the relationship between interpretation bias and post-traumatic growth. In Study 2, cognitive restructuring explained a significant amount of variance in the relationship between post-traumatic stress and post-traumatic growth, with deliberate rumination moderating the effects of cognitive restructuring on post-traumatic growth. Conclusions: Cognitive mechanisms are key to understanding the relationship between post-traumatic stress and growth and should be targeted in interventions to improve cognitive flexibility and resilience among breast cancer survivors.

## Simple Summary

Breast cancer is a life-threatening and traumatic experience that leaves women with distress and anxiety for many years. However, research shows it is possible to grow from trauma in meaningful and adaptive ways. The current investigation attempted to identify the cognitive mechanisms underlying trauma and growth, which are involved in resilience and adaptation. Three important findings emerged. First, we found that the experiences of growth and trauma were negatively related to each other. Second, cognitive strategies such as the ability to restructure the negative impact of trauma in positive and adaptive ways were instrumental in explaining the relationship between trauma and growth. Third, constructive reflections on the past can facilitate the experience of growth in positive ways. Our results suggest that to promote post-traumatic growth in survivors of breast cancer, interventions should focus on empowering individuals with strategies that emphasize cognitive flexibility.

## 1. Introduction

Breast cancer is the most prevalent cancer worldwide and the largest cause of malignancy in women [1,2,3]. Advances in medical treatments have helped increase survival rates, but the psychological costs of breast cancer remain high [4]. Self-reported distress has been linked with a 46% increased risk of mortality due to breast cancer [5], and meta-analyses have shown that anxiety and depression can increase the risk of mortality by up to 30% [6]. A substantial proportion of women can continue to experience numerous side effects of diagnosis and treatment for several years post-diagnosis, impairing quality of life [7,8]. Ongoing stressors, such as fear of recurrence, scan anxiety, pain, fatigue, and cancer-related cognitive decline, can render breast cancer a chronic condition with continued traumatic experiences [9,10,11]. These have pressing implications for reduced quality of life [12,13], impaired workability [14,15,16,17], and reduced self-esteem [18].

Post-traumatic stress symptoms (PTSS) are prevalent among survivors of breast cancer [19], with the lifetime prevalence of symptoms of PTSS in survivors of cancer estimated at 30–40% [19,20,21]. These figures may indeed be an under-estimation, as cancer patients can be under pressure to ‘stay positive’ by friends and family and thus minimise reports of distress [22]. There is a greater risk of developing post-traumatic stress disorder (PTSD) in cancer survivors compared with the general population, with women and younger individuals most affected [23]. Indeed, cancer-related intrusions and unintentional thoughts about cancer tend to interfere with everyday cognition and emotional well-being in breast cancer survivors [24].

Ongoing cancer-related cognitive impairment is an unaddressed problem that can linger for years after active treatment for primary breast cancer [25]. Impairments in working memory and attention, as well as reduced concentration and learning, are well documented [26,27,28]. It is key to address cognitive deficits as impairments in processing efficiency are risk factors for anxiety and depressive symptoms [29,30]. Some evidence suggests that adaptive cognitive training interventions, which boost processing efficiency and working memory performance, can help reduce anxiety and depressive symptoms in survivors of breast cancer [26,31].

Alongside the experiences of post-traumatic stress, studies have found that approximately half of survivors of different types of traumas report post-traumatic growth [32]. Post-traumatic growth (PTG) refers to the cognitive and emotional processes involved in cognitive restructuring, finding meaning, and re-staging priorities in an adaptive way [33]. While growth may not result from the trauma itself, engaging in cognitive restructuring with positive reframing, changing and revaluing priorities, and finding new opportunities can facilitate a greater appreciation for life and growth [34].

In survivors of breast cancer, PTG frequently occurs, with reports of early and late onset after diagnosis [35]. However, personal growth can depend on several cognitive and emotional factors, which involve significant adjustments to the challenges faced in survivorship. Systematic reviews and meta-analyses have demonstrated the importance of considering PTSD [21,36] and PTG [37,38] among breast cancer survivors. However, significant research gaps remain regarding quantitatively investigating the role of cognitive factors in PTSD and PTG among survivors of breast cancer.

The relationship between PTSS and PTG has not been easy to establish. At times, the stage of cancer has been a moderator of this relationship, with a positive relationship seen in stage 4 (incurable) cancer survivors [39]. Studies reporting a negative correlation between stress and growth [40,41] emphasize the adaptive value of growth in reducing distress. Finally, recent reviews have suggested that PTSS and growth can co-occur with stress responses, such as hypervigilance and anxiety regarding one’s health, triggering growth responses including self-care and self-compassion [42]. However, it is noteworthy that not everyone who experiences PTSS will develop PTG, and factors influencing this relationship are yet to be substantiated.

### Research Aims

In two studies, we investigated cognitive mechanisms that can explain the relationship between PTSS and PTG in survivors of primary breast cancer. Cognitive biases such as attentional and interpretation biases for negative information are known to play an important role in the development and maintenance of PTSD [43] and, as such, have been the focus of cognitive therapies. In cancer, negative attentional bias has been associated with PTSS, while positive attentional bias has been associated with PTG [44], suggesting that attentional processes play an important role in predicting stress and growth responses in cancer survivors. Similarly, cognitive restructuring of trauma-related experiences can help re-elaborate the experience of trauma into positive life changes, permitting growth [45].

The two studies reported in this paper were conducted in Iran. In 2021–2022, the World Health Organisation [46] emphasized the need to promote more research into improving the outcomes of cancer treatment programs in countries such as Iran, where rates of breast cancer are increasing, especially in younger women. Despite improved survival rates, psychological distress remains a recognized but unmet public health need in Iran [47] with implications for workability, personal and family health, as well as survival outcomes.

## 2. Study 1

Study 1 investigated the role of positive interpretation bias. Interpretation bias, which is a tendency to interpret ambiguous information in a threatening manner, was investigated among breast cancer survivors, where individuals with higher levels of anxiety interpreted ambiguous information in a negative way, suggesting susceptibility to illness preoccupation [48]. Interpretation bias has also explained the relationship between reported somatic symptoms and fear of cancer recurrence [49] as well as predicting resilience in breast cancer survivors [50]. We predicted that positive interpretation bias would be associated with greater growth and fewer symptoms of PTSS.

### 2.1. Method

#### 2.1.1. Participants

In total, 113 women with a primary or secondary diagnosis of breast cancer were recruited from Velaaiat Hospital’s Oncology Unit in Qazvin City, Iran, and were undergoing active treatment at the time of study (see Table 1 for demographics). Our inclusion criteria were a diagnosis of breast cancer. Power calculations indicated that a sample size of 95 participants was required for a small to moderate effect size and an α of 0.05.

#### 2.1.2. Measures

##### PTSS Disorder Checklist-5 (PCL-5)

The PCL-5 [51] is a 20-item self-report measure of PTSD symptoms, with responses scored on a 4-point Likert scale, ranging from 0 to 80. Higher scores indicated more severe symptoms. There is excellent internal consistency (Cronbach’s α = 0.92) for the Farsi version of the PCL-5 [52]. Internal consistency in the current study was good (Cronbach’s α = 0.93).

##### Post-Traumatic Growth Inventory

This is a 21-item questionnaire measuring PTG [33]. It consists of five subscales (relating to others, new possibilities, personal strength, spiritual growth, understanding of life) and responses are scored on 5-point Likert scales, with higher total scores indicating greater post-traumatic growth. Internal consistency for the Farsi version is established (Cronbach’s α = 0.92) [53], and in our study, internal consistency was good (Cronbach’s α = 0.87).

##### Ambiguous Scenarios Test

The ambiguous scenarios test has been designed to assess interpretation bias [54]. In this test, participants are presented with 24 ambiguous scenarios and asked to imagine each situation and rate its pleasantness (on a 9-point Likert scale, with higher ratings indicating greater positive interpretation bias). Each scenario has an emotionally ambiguous ending (e.g., for example: Your best friend convinces you to go on a blind date, and as you sit and wait to meet your date, you think about how it will go). The AST has an internal consistency of 0.82 [55]. Internal consistency in the present study was good (α = 0.91).

##### Functional Assessment of Cancer Therapy-Cognitive Scale Version-3 (FACT-Cog)

The FACT-Cog [56] consists of 37 items measuring perceived cognitive functioning. Items are scored on a 5-point Likert scale, with higher scores indicating better cognitive function. The FACT-Cog has excellent internal consistency (Cronbach’s α = 0.97) among Iranian samples [57]. In our study, internal consistency was good (α = 0.82).

##### Hospital Anxiety and Depression Scale (HADS)

HADS [58] assesses anxiety and depression (over the past seven days) and has been used widely in breast cancer research. Seven items measure anxiety, and seven items measure depression, with all items scored on 4-point Likert scales with higher scores indicating greater anxiety and/or depressive symptomatology. Scores of 11 and greater on either scale are considered to reach the clinical threshold for the disorder. The Farsi version of the HADS has been validated with good internal consistency (α > 0.70) [59]. In our study, internal consistency was good (Cronbach’s α = 0.93).

#### 2.1.3. Procedure

Ethical approval was sought and approved by The Institute for Cognitive Science Studies, Iran (IR.UT.IRICSS.REC.1401.026). Participants first completed a questionnaire measuring demographic information. After this, participants completed the self-reported questionnaires.

#### 2.1.4. Data Analysis Plan

Data were analyzed using SPSS 28. Correlations were used to investigate the relationships between the variables. A moderated mediation analysis using Hayes Macro PROCESS Model 14 was performed to examine the role of interpretation bias (mediator) in explaining the relationship between PTSS and PTG. The moderating role of perceived cognitive functioning (FACT-COG) was examined in relation to positive interpretive bias (PIB) and post-traumatic growth (PTG). The moderated mediation model specified in this study was grounded using a priori theoretical models, which have established a strong causal role of cognitive biases and appraisals in PTSD [44]. However, given the cross-sectional nature of our study, causality will not be inferred from our analysis. Given the lack of studies exploring the potential mediation pathways in the PTSS–PTG relationship, our study provides a step-change informative framework for future studies.

### 2.2. Results

Table 1 shows the demographics and clinical background for all participants. Descriptive statistics for participant responses on all the questionnaire measures are presented in Table 2. Fifty-six participants (49.56%) scored above the clinical cut-off for PTSD (>33) on the PCL-5. Forty-one participants (36.28%) scored above the clinical cut-off for depression (>10), and 48 participants (42.48%) for anxiety (>10). Sixty-six participants (58.41%) of the sample reported medium to high levels of PTG.

Positive interpretation bias correlated positively with perceived cognitive functioning and PTG (both r > 0.55, both *p* < 0.001) and negatively with anxiety, depression, and PTSS (all r > −0.45, all *p* < 0.001). The correlation between PTSS and PTG was negative, r = −0.58, *p* < 0.001. Age correlated negatively with post-traumatic stress, anxiety, and depression (all r > −0.25, all *p* < 0.01).

**Figure 1:**
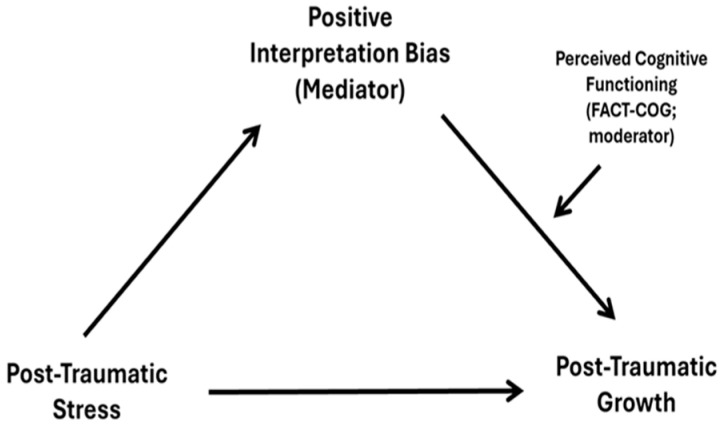
Moderated mediation model with positive interpretation bias as mediator and perceived cognitive functioning as moderator.

**Figure 2:**
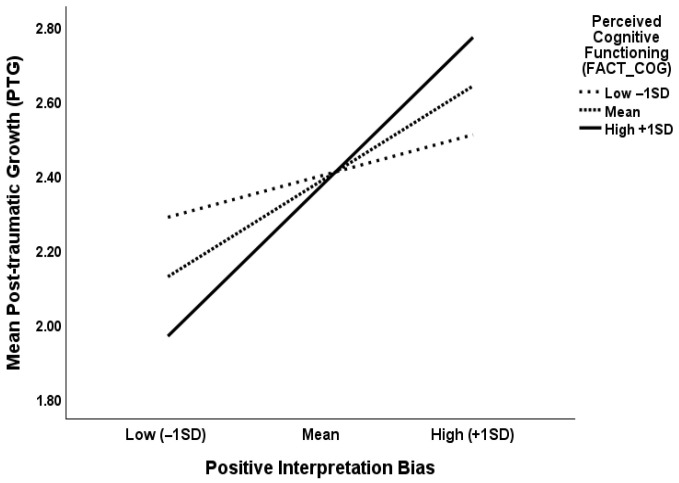
Line graph depicting the moderating effect of perceived cognitive functioning on positive interpretation bias and post-traumatic growth.

The analysis of the moderated mediation model (Figure 1) with positive interpretation bias (PIB) as mediator and perceived cognitive functioning (FACT-COG) as moderator revealed a significant indirect effect of post-traumatic stress symptoms (PTSS) on post-traumatic growth (PTG) through positive interpretation bias (PIB) at medium and high levels of perceived cognitive functioning (FACT-COG). Table 3 shows the unstandardized parameter estimates with bootstrapping results for this analysis. This indicated that the effect of PTSS on PTG decreased through positive interpretation bias with higher levels of perceived cognitive functioning, indicating a protective effect. The analysis of simple slopes shows the interaction of PIB x FACT-COG at low, medium, and high levels of both variables in Figure 2.

In sum, we found a negative relationship between PTSS and PTG. In support of our hypothesis, positive interpretation bias mediated the relationship between PTSS and PTG, with perceived cognitive functioning moderating the relationship between positive interpretation bias and PTG at medium and higher levels of this variable, suggesting that the combined effect of higher levels of perceived cognitive functioning and positive interpretation bias predicted the highest levels of PTG. Our results are suggestive of the protective effect of positive interpretation bias and perceived levels of better cognitive functioning on the effects of PTSS on PTG.

## 3. Study 2

In an extension of Study 1, Study 2 used the cognitive restructuring of trauma measure [60], which assesses both positive (i.e., acceptance and downward comparisons) and negative cognitive (i.e., denial and regret) restructuring. Cognitive restructuring of trauma involves integrating cognitions with existing beliefs, which can include creating new meaning and positive adjustment, adaptation, and acceptance [33,34]. We predicted that positive cognitive restructuring of trauma would explain significant variation in the relationship between PTSS and PTG. Additionally, we were interested in the possible role of rumination as a moderator in the relationship between positive cognitive processing and PTG. Rumination has been considered a cognitive transdiagnostic factor for depression and post-traumatic stress [61] and involves repetitive cycles of unwanted thoughts, usually negative in nature. While intrusive rumination may take the form of automatic unwanted thoughts that are uncontrollable, deliberate rumination (also known as reflective or intentional) is a constructive form of rumination that aims to provide solutions to the experienced trauma [62]. In cancer survivors, deliberate rumination has been shown to predict PTG [63], while intrusive rumination has been associated with and can sustain PTSS [64]. In investigating the roles of intrusive and deliberate rumination in our study, we predicted that intrusive rumination would be associated with PTSS and deliberate rumination would be associated with PTG.

### 3.1. Method

#### 3.1.1. Participants

Participants were Iranian women with a history of breast cancer (*N* = 117). Participants were selected by a convenience sampling method and recruited through social media platforms (e.g., such as Instagram, WhatsApp, and Telegram). Inclusion criteria stated that participants were at least 3 months post-diagnosis of breast cancer and aged over 16 years. Power calculations for a moderate effect size, an alpha of 0.05, estimated a sample size of 100 participants.

#### 3.1.2. Materials

##### Expanded Version of the PTG and PTD Inventory (PTGDI-X-50)

Post-traumatic growth (PTG) was measured using the Expanded version of the PTG and PTD Inventory (PTGDI-X-50) index, which includes 50 items assessing five aspects of post-traumatic growth. Participants rated their experiences on a 6-point Likert scale, where 0 = “I did not experience this change as a result of my crisis” and 5 = “I experienced this change to a very great degree as a result of my crisis.” Total scores for post-traumatic growth range from 0 to 125, with higher scores indicating greater growth. The internal consistency of the index has been high, with a Cronbach’s alpha of 0.93 [65]. In our study, the Cronbach’s alpha was good (α = 0.94).

##### Event-Related Rumination Inventory (ERRI)

The ERRI is a 20-item questionnaire assessing intrusive rumination (10 items) and deliberate rumination (10 items). Items are scored from 0 (never) to 3 (always), with total scores ranging from 0 to 60 and higher scores indicating higher levels of rumination [64]. Internal consistency has been found to be good for both intrusive rumination and deliberate rumination (both Cronbach α > 0.85) [66]. In the current study, internal consistency was good for both intrusive and deliberate rumination (α > 0.85).

##### Cognitive Processing of Trauma Scale (CPOTS)

CPOTS [60] is a 17-item questionnaire measuring positive cognitive restructuring (positive cognitive restructuring, downward comparison, and acceptance) and negative cognitive restructuring (denial and regret). All items utilized a 5-point Likert scale (−3 strongly disagree to +3 strongly agree). Psychometric properties of the Persian version of the CPOTS have been shown to be good [67]. In our study, internal consistency was good for both positive and negative cognitive restructuring of trauma (both Cronbach α > 0.80).

#### 3.1.3. Procedure

Ethical approval was sought and approved by The Institute for Cognitive Science Studies, Iran (IR.UT.IRICSS.REC.1401.011). After providing informed consent, participants completed a demographics questionnaire, which was followed by the self-reported questionnaires PTGDI-X-50, PCL-5 (as described in Study 1), ERRI, CPOTS, and FACT-Cog (as described in Study 1). Data collection was online via the Porsline website (https://porsline.ir/online-questionnaire/ (accessed on 1 December 2022)).

#### 3.1.4. Data Analysis Plan

Data was analyzed using SPSS 28 MACRO Process Model 14. In line with reservations noted for interpretation of causality (as noted in Study 1), our moderated mediation analysis examined the effect of cognitive restructuring of trauma (mediator) on the relationship between PTSS and PTG, with deliberate rumination as a moderator in relation to cognitive restructuring and post-traumatic growth.

### 3.2. Results

Participant characteristics and descriptive statistics for questionnaire responses are presented in Table 4 and Table 5, respectively. Sixty-one participants (52.14%) scored above the clinical cut-off (score ≥ 33) on the PCL-5 [51]. Sixty-eight participants (68.37%) reported medium to high levels of post-traumatic growth.

Positive cognitive restructuring correlated positively with post-traumatic growth, r = 0.68, *p* < 0.001, and negatively with intrusive rumination, perceived cognitive functioning (higher scores reflect poorer functioning), and PTSS symptoms, all r > −0.47, all *p* < 0.001. Negative cognitive restructuring correlated positively with perceived cognitive functioning and PTSS, all r > 0.20, all *p* < 0.02, but not PTG. Deliberate rumination correlated with PTG, r = 0.28, *p* < 0.02, and age, r = −0.28, *p* < 0.01. Intrusive rumination correlated negatively with PTG and positive cognitive restructuring, all r > 0.35, all *p* < 0.02, and positively with perceived cognitive functioning and PTSS (rs > 0.4, ps < 0.001).

**Figure 3:**
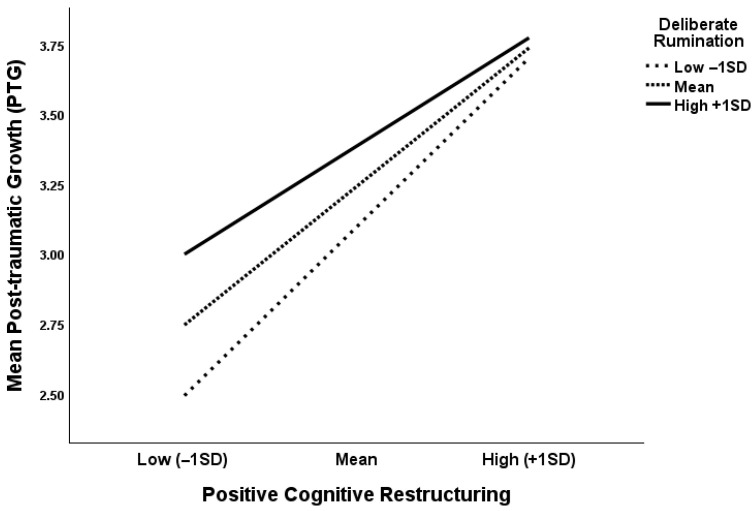
Line graph depicting the moderating effect of deliberate rumination on positive cognitive restructuring and post-traumatic growth (PTG).

A moderated mediation analysis with positive cognitive restructuring (PosCog) as mediator and deliberate rumination (DelRum) as moderator resulted in a significant indirect effect of post-traumatic stress symptoms (PTSS) on post-traumatic growth (PTG) through positive cognitive restructuring at low, medium, and high levels of deliberate rumination (DelRum). Table 6 shows the unstandardized parameter estimates for the effects of interest. The analysis showed that the effect of PTSS on PTG decreased through positive cognitive restructuring (PosCog) at low, medium, and high levels of deliberate rumination (DelRum). These results indicate that deliberate rumination can in combination with positive cognitive restructuring have a beneficial effect on post-traumatic growth. The analysis of simple slopes in Figure 3 shows this effect at low, medium, and high levels of both variables. No significant effects were found for intrusive rumination as a moderator.

In sum, Study 2 also found a significant negative relationship between PTSS and PTG. Our results show that positive cognitive restructuring of trauma mediated the relationship between PTSS and PTG. On the other hand, negative processing of trauma did not explain a significant variation in the association between PTSS and PTG. Our results also show that deliberate, but not intrusive, rumination moderated the relationship between positive restructuring and growth, with deliberate rumination exerting a protective effect in combination with positive cognitive restructuring on PTG.

## 4. General Discussion

Understanding the nature of the relationship between trauma to growth is key to helping survivors of breast cancer thrive in survivorship. Cancer can leave many individuals emotionally and cognitively vulnerable, and this can obstruct PTG by re-instating traumatic stress-related symptoms. As such, it is imperative that we understand the possible pathways that can promote growth and resilience in survivors. Growing from trauma in positive ways has been associated with better well-being, resilience, and adaptation, seeking new opportunities, meaning making, and re-prioritising, all of which imply cognitive flexibility. Importantly, however, elucidating the cognitive factors that can facilitate growth from traumatic stress in cancer is yet to be understood.

In two studies, we have demonstrated that cognitive mechanisms can play a key role in explaining the stress-growth relationship in survivors of breast cancer. In both studies, there was a negative and significant relationship between PTG and PTSS. Study 1 specifically found that interpretation bias, a cognitive mechanism that correlates with growth [44], may play a mechanistic role in the stress–growth relationship [68] and, as such, has significant implications for cognitive mechanisms to promote growth and reduce PTSS in survivors of breast cancer. Extending previous findings on the role of interpretation bias in explaining the relationship between somatic symptoms and fear of cancer recurrence [49], we emphasize the role of interpretation bias in facilitating personal growth and positive adaptation, which are negatively related to anxieties concerning cancer recurrence [37,38].

Additionally, our analyses showed how the combined effects of higher levels of perceived cognitive functioning (indicative of better cognitive functioning) and positive interpretation bias played a protective role in reducing the maladaptive effects of PTSS on PTG. Self-reported cognitive deficits are widely documented in breast cancer survivors and are predictive of anxiety and depressive related symptoms in this population (see [31,69]). Our findings suggest that improving cognitive functioning can be critical in promoting growth from trauma and, as such, should be a major focus in clinical interventions aiming to reduce PTSS and promote PTG.

Study 2 extended the findings of Study 1 by using a different cognitive measure: cognitive restructuring. In this study, positive cognitive restructuring of trauma, a cognitive skill involved in acceptance, re-prioritising, and seeking new opportunities, significantly explained the variation in the relationship between PTSS and PTG. This finding indicates that cognitive skills involved in flexibility and resilience can play an important role in understanding the relationship between stress and growth. On the other hand, negative processing of trauma did not mediate the association between PTSS and PTG, indicating the development of PTG may be more dependent upon cognitive restructuring skills, which involve the practice of positive adaptation. Our results also showed that deliberate, but not intrusive, rumination moderated the relationship between positive restructuring and growth. Intrusive rumination has played a clear role in the maintenance of PTSD [70] and is distinct from deliberate rumination, which seems to be more closely related to cognitive restructuring of trauma [71]. Deliberate rumination involves thinking about the meaning and possible causes and ways of dealing with the trauma, whereas intrusive rumination involves the tendency to activate the trauma without meaning [72]. Deliberate rumination shortly after a traumatic experience has been shown to predict PTG [73] and seems to be independent of the type of trauma experienced [74]. Deliberate rumination has been suggested as an adaptive process involving ‘cognitive work’, ‘reflection’, and ‘re-evaluation’ that has a key role in changing beliefs and schemas about oneself and, as such, can lessen the impact of trauma. Together, the studies suggest that the practice of deliberate rumination should be encouraged, and cognitive biases and perceptions of cognitive functioning should be targeted for promoting PTG in survivors of breast cancer.

Our data suggested that participants were experiencing significant levels of PTSS, with around half of each sample scoring above the clinical cut-off. Additionally, in both studies, over half of the sample reported moderate to high levels of PTG. This indicates that positive and negative post-traumatic psychological outcomes appear common among women diagnosed with breast cancer in Iran, highlighting the need for cognitive interventions guiding the development and implementation of breast cancer-related treatments in Iran [47]. Additionally, Study 1 found age was significantly associated with PTSS, anxiety, and depression, with younger age being associated with greater symptom severity, as well as with anxiety and depression. This suggests that younger women are at a greater risk of post-traumatic stress disorder, and their needs should be a focus of future interventions.

Positive interpretation bias was negatively related to PTSS and positively associated with PTG. According to prominent models of PTSD, interpretation bias of ambiguous cues can perpetuate maladaptive appraisals of ambiguous events, which can maintain stress symptoms [44]. Our findings extend past research in the breast cancer population by documenting a negative relationship between positive interpretation bias and PTSS [75]. Similarly, theoretical models of PTG maintain that cognitive processing styles (and biases) can facilitate positive or negative cognitive changes, which play a defining role in the formation of PTG [76]. Our findings support previous research by demonstrating a positive relationship between positive interpretation bias and growth in breast cancer [45], but also extend this work by demonstrating that positive interpretation bias can explain significant variation in the relationship between PTSS and PTG in the context of breast cancer. In support of models of PTG, our findings suggested that positive cognitive restructuring can be a key factor in the development of resilience and growth by assisting in the re-building of schema promoting meaning-making, adaptation, and resilience (e.g., [76,77]). As predicted, positive cognitive restructuring was negatively associated with PTSS. This finding supports the notion that PTSS can be fueled by negative cognitive restructuring of the trauma and a cognitive mismatch between the trauma and one’s meaning structures [78,79].

Deliberate rumination was positively associated with PTG, which aligns with current theory and research. Deliberate rumination is a crucial determinant of PTG, as it enables individuals to consciously reflect on their experience, find meaning, integrate the experience into their life narrative, and re-evaluate current circumstances [80]. Deliberate rumination was not significantly associated with PTSS, however, which implies that the processes underlying deliberate rumination may not be of a nature that reinforces the trauma and its negative consequences, which is the case for intrusive rumination. Intrusive rumination is considered passive, potentially nonconstructive, and psychologically harmful [81].

### 4.1. Implications for Practice

Our findings provide initial support for treatment targets among women with breast cancer in Iran. The findings support the use of cognitive interventions that promote deliberate rumination and positive cognitive restructuring for the promotion of PTG [82]. Specifically, clinicians should consider assessing interpretation biases, current cognitive restructuring practices, and rumination (intrusive and deliberate) in the initial assessment sessions. These aspects should then be included in formulations, given their potential role in the development and maintenance of PTSS and PTG. Interventions should then specifically target deliberate rumination–teaching patients ways in which to consciously reflect on their experiences, thinking about the meaning and possible causes and ways of dealing with the trauma [68] and integrating the experiences into their life narrative [72,77]. Alongside this, clinicians could consider working with patients to increase their skills in positive cognitive restructuring (such as the ability to revalue, accept, seek new opportunities, and re-prioritise) to re-build schema and promote meaning-making, adaptation and resilience (e.g., [75,77]). This is important as little psycho-oncology research has been conducted in Iran, a country facing an epidemiological transition from communicable to non-communicable diseases [83,84]. The current research responds to an identified Iranian public health priority to improve the understanding of cancer and psycho-oncology treatment [85] and the need for tailored cancer treatment programs to be designed and implemented within Iran.

### 4.2. Limitations and Future Directions

There are several limitations to the current investigation. First, the cross-sectional design limits the causal inferences. Despite the mediation models grounded in a priori theoretical frameworks, the mediation aims and hypotheses were atemporal and thus, causality cannot be inferred from the statistical analyses [86]. Rather, the two studies are important as initial studies in Iran, given the concerning lack of psycho-oncology studies in Iran, in exploring potential explanatory pathways that can inform future research. Thus, further longitudinal studies are needed. Second, while positive interpretation biases were investigated using a routinely used questionnaire, future studies could utilize a more trauma-specific measure to assess whether the biases are trauma and threat-related rather than just negative. Third, given that individuals’ self-reports of PTG may be cognitively biased, future research should consider other methods to assess the variables investigated in these studies. Despite these limitations, our findings significantly add to the literature by demonstrating associations between cognitive factors that may promote longer-term growth and resilience in the breast cancer population in Iran. The current research clearly highlights the need for further research focusing on the cognitive mechanisms involved in PTG and PTSS among survivors of cancer, especially in Iran, where, despite psycho-oncology being an identified public health priority [85], there remains limited research in the field.

## 5. Conclusions

In conclusion, our studies investigate the complex interplay between cognitive mechanisms, trauma, and growth among breast cancer survivors in Iran. By identifying the roles of interpretation bias and cognitive restructuring, as well as the moderating effect of deliberate rumination on PTG, we suggest potential pathways for therapeutic intervention aimed at enhancing resilience and well-being in this group of survivors. These insights support the development of cognitive-based approaches that promote positive interpretation biases, encourage constructive cognitive restructuring, and foster deliberate rumination to facilitate post-traumatic growth. The findings also highlight the prevalent psychological challenges, such as PTSS, faced by younger women, emphasizing the need for targeted mental health support in younger populations. Our research contributes to the broader field of psycho-oncology in Iran, addressing a crucial public health priority to improve cancer survivorship outcomes. Future studies should continue to explore cultural and contextual factors affecting cognitive processes in PTG, aiming to refine treatment strategies and support systems for breast cancer survivors in Iran and similar settings.

## Figures and Tables

**Table 1:** Demographic and clinical information for Study 1.

Demographic Variable	*N* = 113
Age–years	*M* = 52.33, *SD* = 11.11
Age at diagnosis–years	*M* = 49.46, *SD* = 10.95
Relationship status (*n*, %)	
Single	25 (22.1%)
Married	87 (77%)
Divorced	1 (0.9%)
Education (*n*, %)	
8 years or less	44 (38.9%)
High school	49 (43.4%)
Undergraduate	17 (15%)
Postgraduate	2 (1.8%)
Other	1 (0.9%)
Employment status (*n*, %)	
Private or public sector employee	9 (8%)
Self-employment	6 (5.3%)
Unemployed	95 (84.1%)
Other	3 (2.7%)
Diagnosis status	
Primary	76 (67.3%)
Secondary	37 (32.7%)
Type of cancer	
Ductal carcinoma in situ-DCIS	1 (0.9%)
Invasive ductal carcinoma-IDC	17 (15%)
Invasive Lobular Breast Cancer	13 (11.5%)
Inflammatory Breast Cancer	13 (11.5%)
Other	68 (60.2%)
Receiving medication for anxiety or depression	
Yes	13 (11.5%)
No	96 (85%)

Note: DCIS = ductal carcinoma in situ; IDC = Invasive Ductal Carcinoma.

**Table 2:** Descriptive statistics for questionnaires in Study 1.

Study Variables	*M* (*SD*)	Min	Max
Symptoms			
PTSD Symptoms	32.53 (17.30)	5.00	68.00
Anxiety Symptoms	16.91 (10.22)	0	20.00
Depression Symptoms	9.18 (5.17)	0	20.00
PTG	48.56 (14.64)	11.97	74.97
Ambiguous Scenarios Test—Clarity	12.00 (1.54)	7.00	14.00
Ambiguous Scenarios Test—Pleasantness	14.51 (2.65)	7.00	18.00
FACT-COG	98.78 (20.19)	46	134

Note: PTSD = Posttraumatic stress disorder; PTG = Posttraumatic Growth; FACT-COG = Functional Assessment of Cancer Therapy-Cognitive Scale Version-3.

**Table 3:** Moderated mediation analysis of the indirect effect of post-traumatic stress symptoms (PTSS) on post-traumatic growth (PTG) through positive interpretation bias (PIB), conditional on perceived cognitive functioning (FACT-COG). Bootstrap confidence intervals are based on 5000 resamples.

Path	Effect	SE	95% Bootstrap CI
a (PTSS → PIB)	−1.00	0.08	[−1.17, −0.85]
b (PIB → PTG)	0.01	0.003	[0.004, 0.18]
c′ path (PTSS → PTG, direct)	−0.01	0.006	[−0.02, 0.001]
Interaction (PIB x FACT_COG → PTG)	0.18	0.008	[0.003, 0.036]
Indirect effect (FACT_COG = Low, −1 SD)	−0.005	0.005	[−0.15, 0.001]
Indirect effect (FACT_COG = Mean)	−0.01	0.004	[−0.18, −0.004]
Indirect effect (FACT_COG = High, +1 SD)	−0.017	0.004	[−0.24, −0.01]

**Table 4:** Demographic and clinical information for Study 2.

Demographic Variable	*N* = 117
Age—years	*M* = 43.95, *SD* = 8.40
Age at the time of diagnosis—year	*M* = 41.09, *SD* = 7.90
Relationship status (*n*, %)	
Single	14 (10.1%)
Married	95 (68.8%)
Divorced	8 (5.8%)
Education (*n*, %)	
8 Years or less	8 (5.8%)
Diploma or college	30 (21.7%)
Undergraduate	55 (39.9%)
Postgraduate	24 (17.4%)
Employment status (*n*, %)	
Private or public sector employee	21 (15.2%)
Self-employment	15 (10.9%)
Studying	3 (2.2%)
Unemployed	61 (44.2%)
Other	17 (12.3%)
Diagnosis status (*n*, %)	
Primary	102 (73.9%)
Secondary	15 (10.9%)
Type of cancer (*n*, %)	
Ductal carcinoma in situ—DCIS	10 (7.2%)
Invasive ductal carcinoma—IDC	31 (22.5%)
DCIS and IDC Mixed	6 (4.3%)
Invasive Lobular Breast Cancer	14 (10.1%)
Inflammatory Breast Cancer	2 (1.4%)
Other	54 (39.1%)
Grade (*n*, %)	
Grade I	20 (14.5%)
Grade II	59 (42.8%)
Grade III	38 (32.5%)
Type of therapy (*n*, %)	
Chemotherapy	8 (6.8%)
Radiotherapy	1 (0.9%)
Mastectomy	5 (4.3%)
Lumpectomy	2 (1.7%)
Chemotherapy + Mastectomy	10 (8.5%)
Chemotherapy + Radiotherapy + Mastectomy	44 (37.6%)
Chemotherapy + Radiotherapy + Lumpectomy	24 (20.5%)
Radiotherapy + Mastectomy	1 (0.9%)
Chemotherapy + Radiotherapy + Mastectomy + Lumpectomy	4 (3.4%)
Other	18 (15.4%)

Note: DCIS = ductal carcinoma in situ; IDC = Invasive Ductal Carcinoma.

**Table 5:** Descriptive statistics for questionnaires in Study 2.

Study Variables	*M* (*SD*)	Min	Max
PTGDI-X-50	3.23 (0.96)	0.40	4.84
FACT-COG	92.28 (28.48)	10.00	135.00
Intrusive rumination	17.03 (7.18)	3.00	30.00
Deliberate rumination	21.16 (6.34)	6.00	30.00
Positive cognitive restructuring	4.32 (1.16)	0.44	6.00
Negative cognitive restructuring	3.09 (1.14)	0.83	6.00

Note: PTGDI-X-50 = Expanded version of the PTG and PTD Inventory; FACT-COG = Functional Assessment of Cancer Therapy-Cognitive Scale Version-3.

**Table 6:** Moderated mediation analysis of the indirect effect of post-traumatic stress symptoms (PTSS) on post-traumatic growth (PTG) through positive cognitive restructuring (PosCog), conditional on deliberate rumination (DelRum). Bootstrap confidence intervals are based on 5000 resamples.

Path	Effect	SE	95% Bootstrap CI
a (PTSS → PosCog)	−0.035	0.006	[−0.05, −0.02]
b (PosCog → PTG)	0.43	0.074	[0.282, 0.574]
c′ path (PTSS → PTG, direct)	−0.014	0.005	[−0.022, −0.004]
Interaction (PosCog x DelRum → PTG)	−0.015	0.007	[−0.030, −0.001]
Indirect effect (DelRum = Low, −1 SD)	−0.018	0.005	[−0.03, −0.01]
Indirect effect (DelRum = Mean)	−0.015	0.004	[−0.25, −0.008]
Indirect effect (DelRum = High, +1 SD)	−0.012	0.004	[−0.21, −0.004]

## Data Availability

The raw data supporting the conclusions of this article will be made available by the authors, without undue reservation.

## Abbreviations

The following abbreviations are used in this manuscript:

PTSS	Post-traumatic Stress
PTSD	Post-traumatic Stress Disorder
PTG	Post-traumatic Growth
PIB	Positive Interpretation Bias
